# PPG EduKit: An Adjustable Photoplethysmography Evaluation System for Educational Activities

**DOI:** 10.3390/s22041389

**Published:** 2022-02-11

**Authors:** Ángel Solé Morillo, Joan Lambert Cause, Vlad-Eusebiu Baciu, Bruno da Silva, Juan C. Garcia-Naranjo, Johan Stiens

**Affiliations:** 1 Department of Electronics and Informatics (ETRO), Vrije Universiteit Brussel (VUB), 1050 Brussels, Belgium; vlad-eusebiu.baciu@vub.be (V.-E.B.); jstiens@etrovub.be (J.S.); 2 Department of Biomedical Engineering, Universidad de Oriente, Santiago de Cuba 90500, Cuba; 3 Biophysics and Medical Physics Center, Universidad de Oriente, Santiago de Cuba 90500, Cuba; jcgnaranjo@uo.edu.cu

**Keywords:** photoplethysmography, PPG, multi-wavelength, educational platform, analog filters, digital filters, wireless communication, heart rate, blood oxygen level, SpO_2_

## Abstract

The grown interest in healthcare applications has made biomedical engineering one of the fastest growing disciplines in recent years. Photoplethysmography (PPG) has gained popularity in recent years due to its versatility for noninvasive monitoring of vital signs such as heart rate, respiratory rate, blood oxygen saturation and blood pressure. In this work, an adjustable PPG-based educational device called PPG EduKit, which aims to facilitate the learning of the PPG technology for a wide range of engineering and medical disciplines is proposed. Through the use of this educational platform, the PPG signal can be understood, modified and implemented along with the extraction of its relevant physiological information from a didactic, intuitive and practical way. The PPG Edukit is evaluated for the extraction of physiological parameters such as heart rate and blood oxygen level, demonstrating how its features contribute to engineering and medical students to assimilate technical concepts in electrical circuits, biomedical instrumentation, and human physiology.

## 1. Introduction

Nowadays, technological solutions for education are more available than ever. These tools and resources are accessible for both students and teachers and they can range from generic online teaching tools to more specific software and hardware resources. Students acquire practical learning experience through two modes: (1) laboratory exercises integrated into individual courses and (2) laboratory courses developed from a series of experiments. As reported in [1], these practical learning modes are necessary to improve students’ understanding of technical concepts acquired through lectures. These practical learning environments empower students to acquire a leading role, making necessary to promote an active learning process in the construction of their knowledge. Moreover, a recent study [2] has evaluated the effect of project-based learning and flipped classroom approaches on students’ outcomes, concluding that they contribute to improving the learning process in all the parameters evaluated. This leads to an improvement in reasoning, increases creativity, the spirit of cooperation and encourages initiative and self-learning, among others. In addition, a better adaptation has been noted when facing various situations in their daily life. Choosing the right educational devices for these practical learning activities is of utmost importance in order to achieve the quality and outcomes expected.

Biomedical engineering combines knowledge of living systems with engineering principles. It has become one of the fastest growing disciplines in the past years [3,4]. The constant evolution of this multi-disciplinary field leads to a high demand of novel resources, including an improvement of the medical technology used in education [5]. The topics covered in biomedical engineering and medical programs include those related to the cardiovascular system. Due to its relevance, a great variety of practical and laboratory sessions are usually derived from this subject which include the acquisition and processing of photoplethysmography (PPG) signals.

The here presented PPG-based educational platform, called PPG Edukit, is designed to be an open source tool. The PPG EduKit is an adjustable PPG system that aims to provide an educational platform where all the different steps to acquire the PPG signal can be understood, modified and implemented along with the extraction of its relevant physiological information from a didactic, intuitive and practical way. By using the PPG EduKit, students can assimilate technical concepts in electrical circuits, biomedical instrumentation, human physiology, embedded systems design, signal processing (including machine learning) and network connectivity. This makes the PPG EduKit an interesting educational tool not only for biomedical engineering and medical disciplines, but also for other engineering domains such as electrical engineering or applied computer science.

The main contributions of this work are:

- New flexible educational platform to understand the PPG signal.
- Ability to observe, interact and adjust different stages involved in the acquisition and processing of the PPG signal.

The work is organized as follows. A relevant description of the background in order to understand the proposed PPG EduKit is done in Section 2 while the related work is discussed in Section 3. In Section 4, a detailed description of the PPG EduKit is provided. The PPG EduKit is evaluated in Section 5 for several popular uses of PPG technology and discussed in Section 6. Finally, some conclusions are drawn in Section 7 about the proposed educational kit.

## 2. Background

### 2.1. Principles of Photoplethysmography

Nowadays, PPG has been adopted as a simple and low-cost optical bio-monitoring technique used to non-invasively measure the blood volume changes that occur in the microvascular bed of tissue in the different skin layers, which are due to the pulsatile nature of the circulatory system [6].

**Figure 1:**
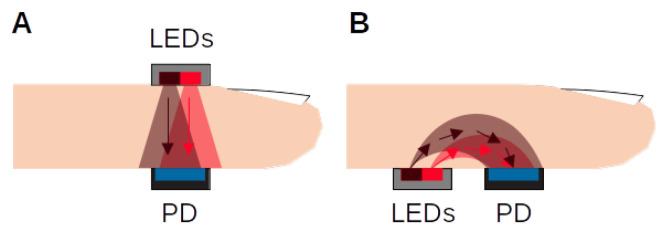
Light-Emitting Diodes (LEDs) and PhotoDetector (PD) placement for transmission (**A**) and reflectance-mode PPG (**B**). Two LEDs (red and infrared) are displayed, a typical configuration in PPG applications.

As an optical technique, PPG requires a light source and a photodetector to operate. The light source illuminates the tissue and the photodetector senses the small variations in light intensity associated with changes in perfusion in the capillary volume. The PPG can be used in two operation modes- transmission and reflection- as shown in Figure 1.

In transmission mode, the light transmitted through the medium is detected by a PhotoDetector (PD) opposite to the Light Emitting Diode (LED) source, while in reflection mode, the PD detects light that is back-scattered or reflected from tissue, bone and/or blood vessels. The transmission mode can obtain good signals, but it can only be used in body sites with limited thickness, such as fingertips, earlobes or nasal septum, in order to be able to detect light. The reflection mode does not have this problem and it enables measurements in other body sites such as wrists, chest or forehead.

The fundamental principle of PPG relies on the higher sensitivity of certain optical wavelengths for blood rather than other tissue components. The ideal wavelength should have a great absorption for blood, allowing accurate monitoring of microvascular changes. Red light (~660 nm) and IR light (~940 nm) are the clinical standard wavelengths used in pulse oximeters for the calculation of oxygen saturation. Green light (~550 nm) is being widely used in reflection-mode applications, since it has greater absortivity for both oxyhaemoglobin and deoxyhaemoglobin than red/IR light [6,7].

**Figure 2:**
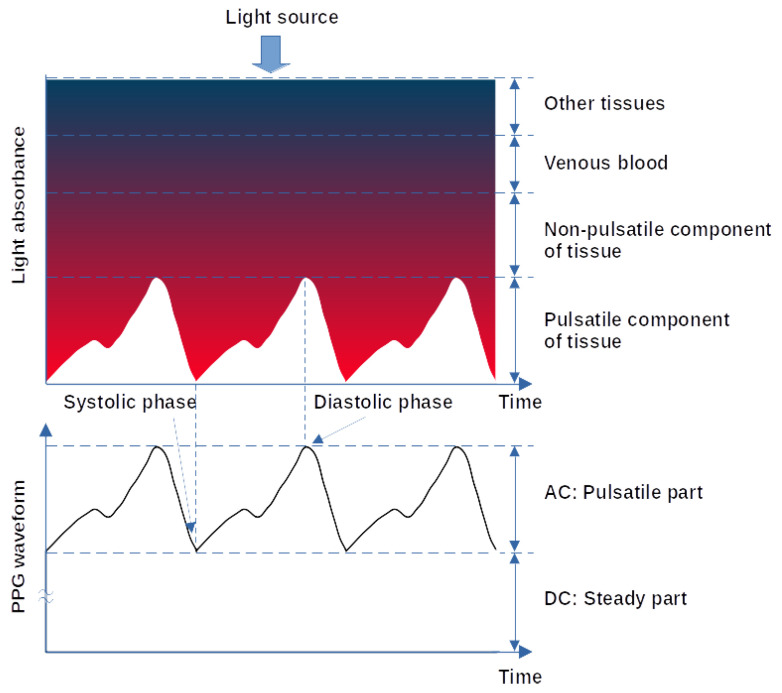
Variation in light attenuation produced by interaction with tissue [6].

When the heart pumps blood to the body and the lungs during systole, the amount of blood that reaches the capillaries in the skin surface increases, resulting in more light absorption. The blood then travels back to the heart through the venous network, leading to a decrease of blood volume in the capillaries and less light absorption. Therefore, the measured PPG waveform comprises a pulsatile (often called ‘AC’) physiological waveform that reflects cardiac synchronous changes in the blood volume with each heartbeat, which is superimposed on a much larger slowly varying quasi-static (‘DC’) baseline (Figure 2). The DC component contains valuable information about respiration, venous flow, sympathetic nervous system activities and thermoregulation [8].

To extract diagnostic information, many features have been investigated including, beat-to beat PPG rise time, pulse transit time (time difference between the R wave peak of the ElectroCardioGram (ECG) signal and the PPG peak), amplitude, shape, and the variability of each of these [9].

### 2.2. Clinical Applications

Photoplethysmography plays an important role in clinical physiological monitoring, since it can measure several vital signs (heart rate, blood pressure and respiration rate), among other parameters, including oxygen saturation. As a result, multiple PPG clinical applications are possible.

#### 2.2.1. Heart Rate

HR helps assessing the cardiovascular system and the diagnosis and detection of coronary diseases; a normal HR range in adults is of 60–100 beats per minute (bpm) [10]. This HR value can be easily obtained with PPG: the light intensity captured by the photo-diode is proportional to the change in blood volume, which is synchronous to the heart beat. Each heart beat can be then linked with the time period of the AC component in the PPG signal. Furthermore, the PPG is an outstanding detector of cardiac arrhythmia, in particular is sensitive to any irregularity of the pulse [11].

#### 2.2.2. Blood Oxygen Saturation

Blood oxygen saturation (SO${}_{2}$) is the fraction of saturated haemoglobin or oxyhaemoglobin (haemoglobin with bound oxygen), relative to total haemoglobin (unsaturated and saturated). Usually, levels of SO${}_{2}$ range from 96 to 99% in healthy individuals.

This oxygen saturation can be estimated by the percutaneous oxygen saturation (SpO${}_{2}$), which can be calculated with PPG by means of a rapid switching between red (~660 nm) and IR (~940 nm) light through vascular tissue [12].

**Figure 3:**
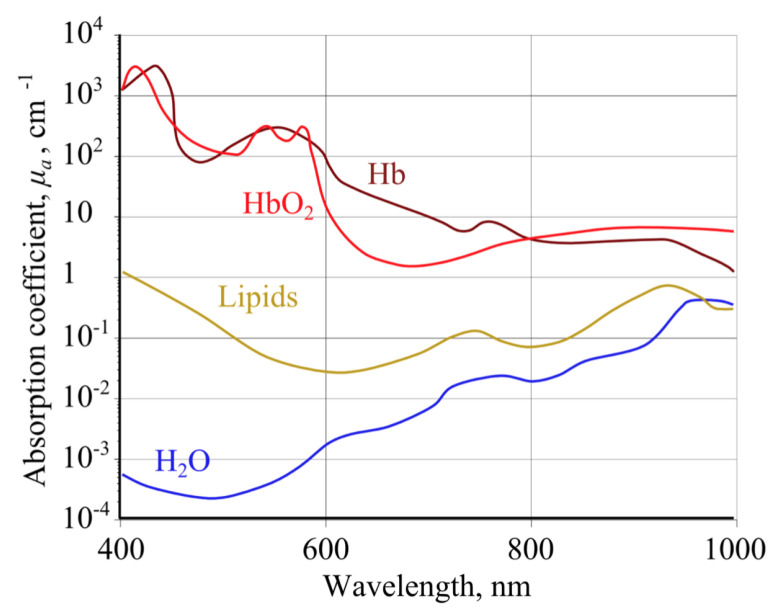
Absorption spectra of oxygenated haemoglobin (HbO_2_) and deoxygenated haemoglobin (Hb) for different wavelengths [13].

As it can be seen in Figure 3, oxyhaemoglobin absorbs less of the red light and more of the infrared, being the opposite for deoxy-haemoglobin. When light at these two wavelengths is emitted in the tissue, the difference in absorption can be exploited to estimate the quantity of oxy- and deoxy-haemoglobin. Therefore, considering the *AC* and *DC* components of both simultaneously-measured PPG signals, SpO${}_{2}$ can be derived from the ratio of absorbances *R* (also known as “ratio of ratios”) (*R*), as follows:

(1) $$R=\frac{{\left(AC/DC\right)}_{red}}{{\left(AC/DC\right)}_{IR}}$$

Once the *R* value is calculated from the two PPG signals, SpO${}_{2}$ values are determined from *R* by using Equation (2):

(2) $$\%{\mathrm{SpO}}_{2}=K\times R$$

where *K* is the proportionality constant, obtained through calibration results [14].

#### 2.2.3. Others

PPG can be further exploited to estimate additional physiological parameters.

Respiration causes variation in the peripheral circulation, making it possible to monitor breathing using a PPG sensor attached to the skin [8,15,16].

Recent studies including multi-site PPG together with machine learning and artificial intelligence techniques, have also enlarged the possibility of using PPG for cuff-less and continuous monitoring of blood pressure [17,18].

Additionally, the analysis of the PPG waveform can assist researchers and clinicians in evaluating various diseases related to the cardiovascular system, such as atherosclerosis, arterial stiffness, and cardiovascular variability. In [8], the author reviews other applications of PPG such as evaluation of endothelial function, venous assessment, vasospastic conditions, microvascular blood flow, tissue viability, autonomic function, vasomotor function, thermoregulation, orthostasis and neurological activity. Recently it has also been used for the detection and clasification of diabetes [19].

### 2.3. Common Signal Conditioning Stages

**Figure 4:**
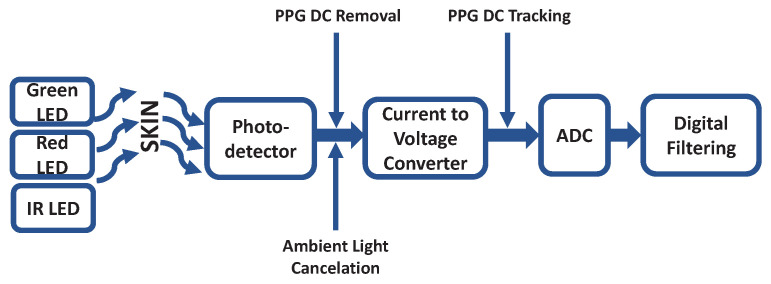
Building blocks depicting the most common signal conditioning stages found in PPG devices in case the application needs the complete PPG signal (AC and DC components).

**Figure 5:**
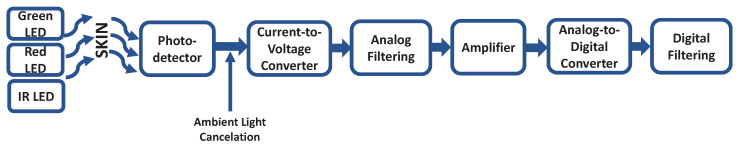
Building blocks depicting the most common signal conditioning stages found in PPG devices if the application only needs the AC component of the PPG signal.

The extraction of the PPG signals needs of common signal conditioning stages which can be seen in a wide range of PPG devices. The signal conditioning operations involve current-to-voltage conversion, filtering, amplification and noise reduction among other operations needed to extract relevant physiological information from the PPG signal. Figure 4 and Figure 5 depict the two main groups of signal conditioning topologies based on the end use of the PPG signal. Those applications requiring both the AC and DC components of the PPG signal, as it is the case in pulse oximeters for SpO${}_{2}$ calculations, mostly use the topology depicted in Figure 4 [20,21,22]. On the contrary, if the application only needs the AC component, the topology depicted in Figure 5 is typically observed [23,24,25].

These topologies have common signal conditioning operations which are here briefly described.

- **Light-Emitting Diodes (LEDs):** PPG applications mainly use green (~550 nm), red (~660 nm) and/or IR light (~940 nm) wavelengths. Red and IR are the clinical standard wavelengths used in pulse oximeters for the calculation of oxygen saturation [6], while the green wavelength is often used in wearables.
- **LED Driver:** The LED activity is normally controlled by a driver, which modulates the LED pulses and current, while the LED pulse widths is typically in the range of a few kHz, the current can go up to 50 mA [20].
- **Photodetector (PD):** Wide spectrum PDs that cover the three wavelengths mentioned above are normally used for PPG measurements. The spectral sensitivity can go from 400 nm to over 1000 nm. The photocurrent generated can range up to 1 μA [26].
- **Current-to-Voltage Converter:** The PD must be followed by a current-to voltage (I-V) converter. The most widely used circuit is a trans-impedance amplifier, where the input current is converted into an appropriate voltage through a resistor that also amplifies the signal. A capacitor can be placed in parallel to the resistor to either stabilize the circuit and/or to form a low-pass filter for the input signal current [27]. There are additional topologies that can be used for the I-V conversion, such as a switched integrator with a capacitor in the feedback loop or a logarithmic amplifier, which uses a diode instead of a capacitor [28].
- **Ambient Light Cancellation (ALC):** Ambient light leakage, i.e., photocurrent generated by ambient light and not by the LEDs occurs during PPG signal acquisition. Many PPG conditioning circuits include an ambient light cancellation module that compensates for this leakage in the form of a current injection or an added voltage before, at or after the I-V converter [22,29]. Ambient light is detected and the compensating current/voltage is sourced by an Digital-to-Analog Converter (DAC).
- **PPG DC Tracking/Removal:** In PPG applications where the physiological DC component of the signal is needed (e.g., SpO${}_{2}$ calculations), such DC must be carefully tracked. Once calculated, the DC component (up to a 80–90% of the PPG signal) can be filtered out allowing for more of the available ADC dynamic range to be used.
- **Analog-to-Digital Converter (ADC):** The PPG signal is an analog signal. In order to be able to treat it digitally, an ADC is needed. PPG applications use ADCs up to 22-bits [29].
- **Analog Filtering:** Many PPG applications use analog filtering modules to filter out undesired noise components. First and second order active band-pass filters are often used to filter DC components (with cutoff frequencies as low as 0.15 Hz) as well as high frequency noise signals (cutoff frequencies from 5 Hz up to 60 Hz) [27,30]. The low-power consumption of these filters makes them ideal for applications where the physiological DC component of the PPG signal is not needed. As previously mentioned, filtering out the DC component allows for more of the available ADC dynamic range to be used. These cutoff frequencies have RC time constants (τ) in the order of a few milliseconds up to 2000 ms. Cutoff frequencies of 0.15 Hz and 6 Hz yield τ values of 1060 ms and 26 ms, respectively. These time constant values are not compatible with SpO${}_{2}$ calculations where much faster LED switching is required.
- **Inverting Amplifier:** An extra amplification stage is commonly placed between the analog filtering stage and before the ADC aiming to further optimize the ADC dynamic range [25,27].
- **Digital Filtering:** Digital filtering is a common practice, especially if no analog filtering stage is present [31,32].

Note that not all applications necessarily integrate all the buildings blocks per topology, nor that the presented arrangements are always strictly followed.

## 3. Related Work

This section comprises an overview of some readily available devices used to extract and study the photoplethysmographic signal. Several representative technologies are categorized depending on the intended use. Whilst most of these devices allow to visualize and store the PPG signal, as well as calculating some physiological parameters (HR and SpO${}_{2}$ mainly), they leave little room for engineering students to understand from an electronics point of view how the signal is acquired and conditioned. Solutions with an educational approach focus on signal processing and feature extraction directly, creating an abstraction layer from the hardware employed for signal conditioning. Other non-educational oriented solutions directly prevent the hardware and software from being disclosed.

From our perspective, existing PPG devices can be categorized in certified medical devices, evaluation systems, DIY (Do It Yourself) solutions and wearables.

### 3.1. PPG in Medical Devices

The pulse oximeter is the only certified medical device that uses PPG, which since the 90’s has been a standard for monitoring during anaesthesia [33]. From the engineering point of view, these commercially available pulse oximeters are ‘black boxes’ where little or no information about the electronics or signal processing is disclosed. Among all the different options available, this study focuses on the evaluation of two widely used pulse oximeters. Nevertheless, their use as reference gold standards when measuring PPG with non-certified devices is highly recommended.

The Masimo MightSat Rx pulse oximeter [34] uses the Masimo Signal Extraction Technology (SET^®^) to measure SpO${}_{2}$, HR, Perfusion Index and Respiration Rate during motion and low-perfusion conditions. While conventional pulse oximetry uses the standard red over IR algorithm to provide SpO${}_{2}$, Masimo SET^®^ uses four other algorithms on top of that conventional algorithm. These algorithms allow the distinction between arterial and venous signal during motion and low perfusion by identifying and isolating the non-arterial and venous noise SpO${}_{2}$ from the true arterial SpO${}_{2}$ components in the signal [35].

The Nonin Onyx Vantage 9590 Finger Pulse Oximeter is indicated for use in measuring and displaying oxygen saturation of arterial hemoglobin and pulse rate of patients who are well or poorly perfused. It uses PureSAT^®^ SpO${}_{2}$ technology, a filtering technique to provide accuracy in the presence of motion or during low perfusion, among others [36].

### 3.2. PPG Evaluation/Development Kits

**Figure 6:**
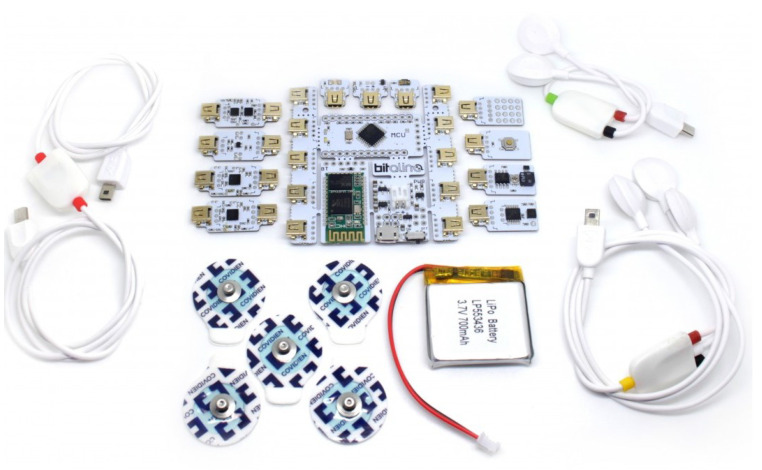
BITalino (r)evolution Plugged Kit BLE/BT. The motherboard with some of the attachable sensors is shown.

The BITalino platform depicted in Figure 6 is an affordable and open-source biosignals platform that allows anyone from students up to professional developers to create projects and applications using physiological sensors [37]. PPG is one of the different biosignals that can be measured with the platform.

**Figure 7:**
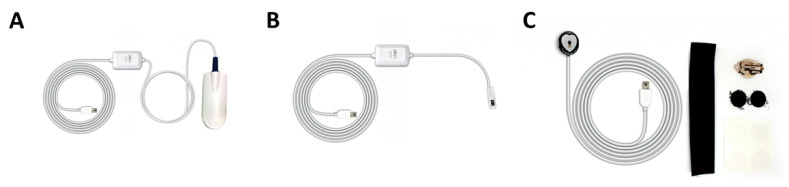
PPG sensors that can be attached to the BITalino platform (**A**) Biosignalsplux SpO${}_{2}$ sensor designed for oxygen saturation level at the finger. (**B**) Biosignalsplux SpO${}_{2}$ sensor designed for oxygen saturation level at multiple body locations. (**C**) Basic sensor for photoplethysmography, it is an adaptation of the PulseSensor.

It can support up to three different types of PPG sensors (Figure 7), where the raw signal together with the HR and SpO${}_{2}$ estimation can be visualized and recorded. Additionally, it also enables the modification of the LED intensity in two of the sensors.

Similarly, the PulseSensor (Figure 7C) is a basic PPG-based heart-rate sensor which can be combined with the Bitalino. It essentially combines a simple PPG sensor (green light) with amplification and noise cancellation circuitry making it fast and easy to get reliable pulse readings [23].

Although Bitalino and PulseSensor are a great educational and professional platform for multi-biosignal analysis, it does not allow the understanding and tuning of all the different building blocks needed for PPG signal acquisition and analysis.

The MAX30101 Evaluation Kit (EV kit) from Maxim Integrated [38] allows the evaluation of the MAX30101 [20], which is an integrated pulse oximetry and heart-rate monitor integrated circuit (IC). The MAX30101 [20] includes internal LEDs (green, red and IR), a photodetector together with low-noise electronics including ambient light rejection and digital filtering.

The MAX30101 EV kit monitors and stores the recorded PPG signals while allowing the user to modify the sampling rate, LED currents and pulse widths. It also offers demo algorithms for HR and SpO${}_{2}$ computation. It is a great tool for professionals, offering a complete system solution to ease the design-in process for mobile and wearable devices that use the MAX30101. Despite this, the evaluation system it is not designed to understand how the PPG signal is acquired but rather to optimize such acquisition.

Another PPG module from Maxim Integrated is the MAXREFDES117 [39]. It features the MAX30102 sensor, which has the same capabilities as the MAX30101, except for a the lack of green light to perform the measurements. It is perfect for DIY wearable projects and it can be placed on a finger or earlobe to accurately detect HR. A basic, open-source heart-rate and SpO${}_{2}$ algorithm is included in the example firmware.

Similarly, the AFE44x0SpO${}_{2}$EVM from Texas Instruments is another kit intended for evaluating AFE4400 and AFE4490 devices. The AFE4490 is a complete analog front-end solution targeted for pulse-oximeter applications. The device consists of a low-noise receiver channel, an LED transmit section, and diagnostics for sensor and LED fault detection. The software of the AFE44x0SpO${}_{2}$EVM includes a GUI that allows the configuration of the I–V amplifier, the ambient light cancellation DAC, the analog filtering, the ADC sampling and LED pulse & intensity, among many other parameters [29]. This is a very complete evaluation system with high adjustable capabilities that, like the MAX30101 EV kit, it is designed for professionals rather than for engineering students. The knowledge entry level is complex for students learning photoplethysmography. Besides, the price is high (190 €) for educational purposes, where multiple units are normally required.

### 3.3. PPG in DIY Solutions

In recent years, the PPG technology has been integrated in DIY projects. Most of these projects are found in the electronic hobbyist community (e.g., Instructables, Hackaday…) where many of them use the PulseSensor module or the MAXREFDES117 described above in combination with an Arduino-like microcontroller [40,41]. The added value of these projects is that they are easy to implement, open source and open hardware. A product description, schematics and a sample code to visualize/store and compute HR/SpO${}_{2}$ are most often provided. This is a great approach for beginners and hobbyist where an introduction to PPG and/or an application is sought. Nevertheless, they tend to be insufficient if a thorough understanding of PPG is desired. The authors believe that similar kits are being used for teaching activities, despite not being published.

### 3.4. Wearables Using PPG

Over the past few years, PPG technology has been widely used in commercial activity trackers, where it is used to calculate HR and SpO${}_{2}$. These activity trackers are mainly wrist-worn and the user can visualize and store HR and SpO${}_{2}$ information without having access to the raw data, while being a good application for the general public, these wearables remain a “black box”, where the conditioning stages and signal processing algorithms reaming out of the user’s reach. Therefore, they are not of the greatest interest for engineering students aiming to understand the PPG acquisition process.

### 3.5. The Need for a New Platform

Up to the authors’ knowledge, there are not platforms oriented to the study and analysis of the PPG signal with a clear educational approach which also allow to flexibly analyze the different stages needed for the signal acquisition. We believe that many of these applications may be used internally for teaching, without being reported in the literature. The here presented work aims to revert this tendency and we hope to see more PPG kits for education published in the literature in the future. The PPG EduKit provides a novel, practical an unique approach to understand and tune the different building blocks needed to obtain the PPG signal as well as the computation of heart rate and SpO${}_{2}$ using different methods.

## 4. PPG EduKit Platform

The PPG EduKit is an evaluation platform aiming to acquire the PPG signal from the finger(s) for research and educational purposes. The PPG EduKit gives the user full control and understanding over the different steps needed to acquire, condition, visualize and process the raw PPG signals which are then used to extract meaningful physiological information. The modular approach of the platform facilitates the support of different embedded devices such as Arduino [42], to perform digital signal processing operations needed for the multiple PPG applications. The components of the PPG EduKit are grouped in two main blocks: the analog front-end and the digital back-end.

### 4.1. Analog Front-End

**Figure 8:**
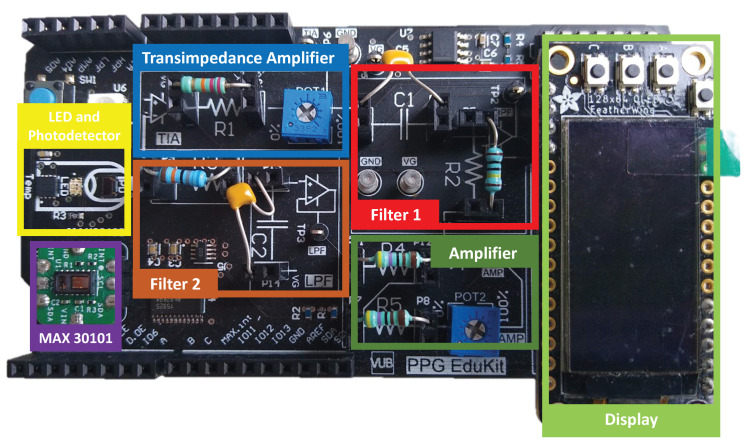
Top view of the PPG EduKit depicting the analog front-end. The most relevant parts are framed and labelled in different colors.

The analog front-end block depicted in Figure 8 is composed of analog components such as filters or passive components (e.g., resistors, capacitors…) and digital components such as the LED driver and temperature sensor. Most of these components are part of the adjustable PPG analog front-end, but additionally, the analog front-end also features a built-in MAX30101 sensor module [20]. This commercial module integrates all the PPG signal conditioning needed to address further educational objectives. As a result, the PPG signals can be acquired independently or simultaneously using either the MAX30101 or the adjustable analog setup.

#### 4.1.1. Adjustable PPG Analog Front-End

**Figure 9:**
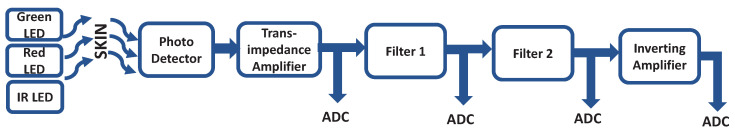
The analog front-end is composed of several signal conditioning stages, including a transimpedance amplifier, analog filtering and an inverting amplifier. The PPG signal can be acquired after each stage.

**Figure 10:**
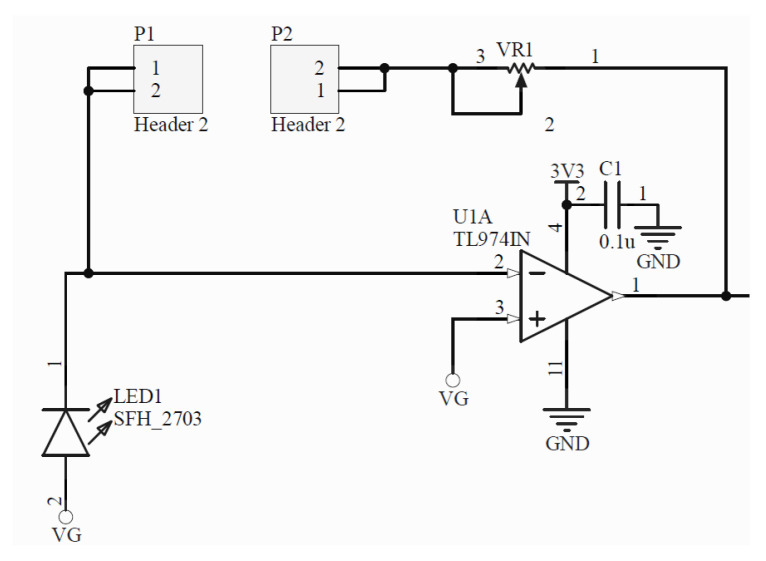
PPG current-to-voltage converter circuit (transimpedance amplifier) with adjustable amplification by means of a through-hole resistor (coarse tuning) and a potentiometer (fine tuning).

**Figure 11:**
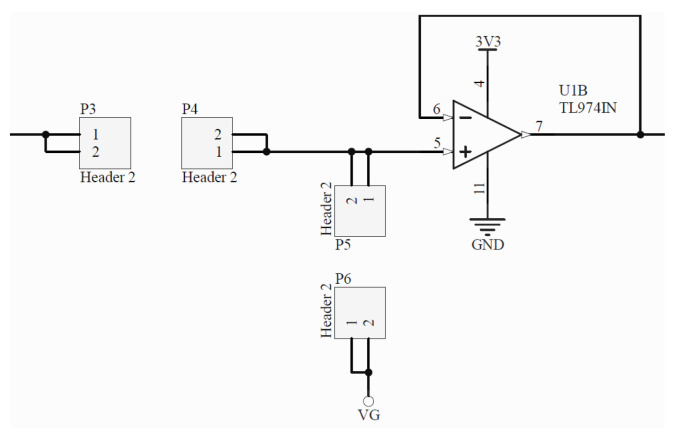
First-order analog filtering circuit with adjustable cutoff frequency values by means of through-hole resistors and capacitors, forming a first-order RC circuit followed by a buffer.

**Figure 12:**
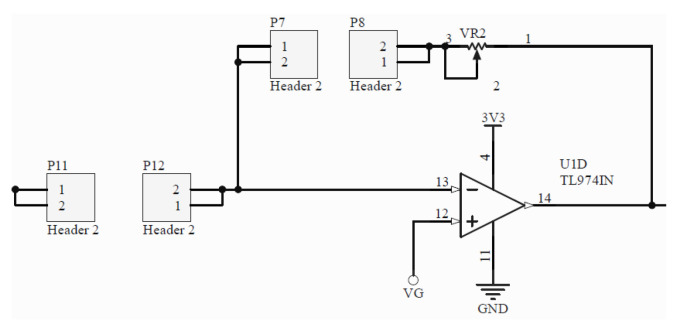
Inverting amplifier circuit with adjustable amplification values by means of through-hole resistors (coarse tuning) and a potentiometer (fine tuning).

The PPG EduKit has an adjustable PPG sensor composed of multiple analog components that can be modified by the user. A system-overview is depicted in Figure 9. All the stages have a direct connection to an analog pin of the digital back-end, as well as analog test points so that each stage can be digitally converted by an ADC and/or visualized with an oscilloscope, respectively. The user can choose from which stage(s) the PPG signal is recorded. The main components of this block are:

- **Multi-wavelength LED and LED driver:** The generation of the optical signal is done using an Osram SFH 7013 IC, which is used as the LEDs source. It includes green (530 nm), red (655 nm) and IR (940 nm) wavelengths, making it ideal for PPG aplications. The SFH 7013 is controlled by the TLC5925 LED driver, which regulates the LEDs pulse and current (up to 60 mA).
- **Photodetector:** The reflected optical information is captured using an Osram SFH2703 photodiode, which has a spectral range of sensitivity of 400 nm to 1100 nm, being able to detect the wavelengths of interest.
- **Transimpedance Amplifier:** The TransImpedance Amplifier (TIA) is a current-to-voltage circuit that convert the current from the photodetector to a voltage for the following stages. As depicted in Figure 10, this component includes an adjustable amplification by setting the right through-hole resistance (coarse tuning) and potentiometer (fine tuning) values.
- **Analog Filtering:** For the analog filtering, the adjustable analog front-end cascades two of the circuits (Filter 1 and Filter 2) depicted in Figure 11. Each circuit consists of a first order RC circuit followed by a buffer. The blocks have 2-pin female jumpers so that through hole components can be connected to form the desired circuit topology. Since the user can choose the RC values, multiple filtering combinations are possible. The most common combination would be a band-pass filter of 0.5–5 Hz, as detailed in Section 2.3. A 50–60 Hz notch filter can be another useful implementation. If the user does not want to use one these modules, i.e., the analog filtering, 0 Ohm resistors will make such blocks simple buffer circuits.
- **Inverting Amplifier:** As depicted in Figure 12, this circuit consists of an inverting amplifier with a potentiometer, which allows the user to control the amplification.
- **Temperature Sensor:** The other digital component is a temperature sensor (MAX30205), which measures the finger temperature. This component also communicates through I2C.

#### 4.1.2. MAX30101

The analog front-end also integrates a MAX30101 module [20]. This commercial module is meant to be used as a standalone sensor or as a comparison with our adjustable PPG analog front-end previously described.

### 4.2. Digital Back End

The PPG EduKit is also composed of a digital back-end. The digital back-end not only complements the analog front-end, but it also provides further support for signal processing operations. These operation can be simple (e.g., analog to digital conversion and wired communication) or more advanced (e.g., wireless communication in combination with advanced signal processing techniques such as machine learning). The support of these operations is determined by the type of the embedded platform considered.

#### 4.2.1. Supported Platforms

The analog front-end interfaces the digital back-end through an Arduino header. The selection of this type of interface is intended to facilitate the use of multiple embedded platforms supporting this type of interconnection while addressing low-cost embedded platforms. Thus, the most appropriated embedded platform can be selected based on the platform cost and the computational needs of the target application.

The supported platforms can be grouped in those one integrating microcontrollers, System-on-Chip (SoC) devices or advance technologies such as Field-Programmable Gate Arrays (FPGAs).

**Figure 13:**
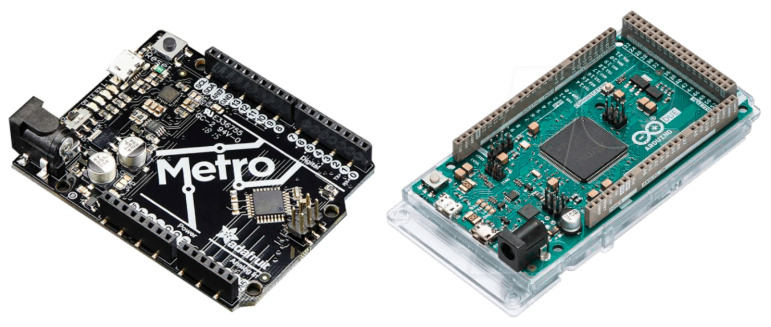
Adafruit Metro (**left**) and Arduino Due (**right**) are two of the Arduino boards compatible with the PPG EduKit.

**Figure 14:**
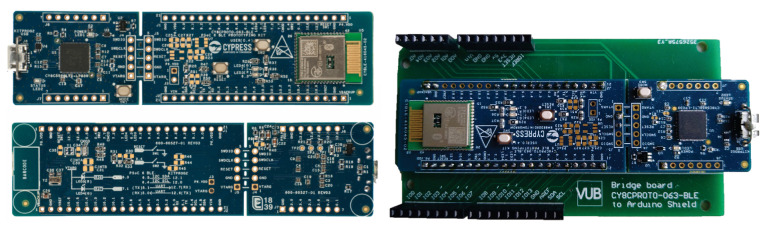
Infineon PSoC 6 BLE Prototyping Kit (**left**) with our bridge board which enables compatibility with Arduino shields (**right**).

**Figure 15:**
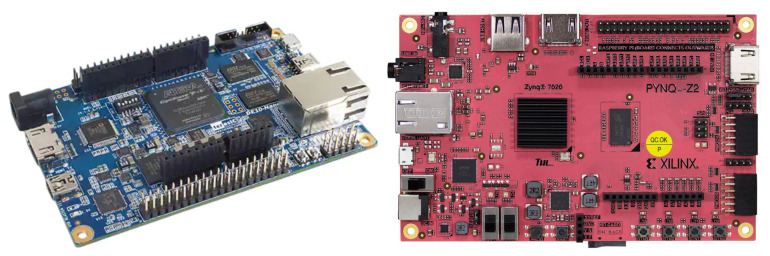
SoC FPGAs compatible platforms. Both Intel DE1-Nano (**left**) and Xilinx PYNQ Platform (**right**) include an Arduino header compatible with the PPG EduKit.

- **Microcontrollers:** The two compatible Arduino boards depicted in Figure 13 are a AdaFruit Metro and an Arduino Due, both integrating microcontrollers Atmel328 microcontroller and an Atmel SAM3X8E, respectively. These platforms are powerful enough to support real-time PPG raw signal filtering while interfacing through I2C the on-board display of the analog front-end. Such platforms are also convenient to introduce the basis of the PPG EduKit due to large Arduino community. Multiple open source libraries can be used to embed the execution of PPG-related applications. Further examples are provided in Section 5.
- **Programmable System-on-Chip (PSoC):** Infineon PSoCs are advanced microcontrollers. These devices include an ARM Cortex-based CPU and mixed-signal arrays of configurable integrated analog and digital components. Such heterogeneous architecture allows the combination of analog operations with digital signal processing, which can be exploited when considering the characteristics of the PPG EduKit. Although there exist several PSoC development platforms supporting Arduino interface, low-cost prototyping boards are compatible with our PPG EduKit. Figure 14 shows how a bridge board can be used to interconnect our PPG EduKit and the CYC6CPROTO-063-BLE board. Although it is not the original goal, the use of such bridge boards extend the list of compatible platforms.
- **FPGAs and SoC FPGAs:** FPGAs are advanced embedded technologies which offer high performance, low latency and power efficiency. Custom architectures can be allocated on FPGAs, leading to a high level of parallel signal processing. This technology is often used for applications demanding fast and power efficient machine learning operations [43]. Figure 15 depicts two FPGA development boards compatible with the PPG EduKit.

#### 4.2.2. Wireless Communication

One of the tasks of the digital back-end is to provide and manage the wireless communication. Bluetooth, Bluetooth Low Energy (BLE) or Wifi are common wireless standards available in most of the commercial embedded platforms. For instance, the low-cost prototype PSoC6 board provides BLE communication. The flexibility of the PPG EduKit enables the use of the more appropriated wireless standard based on the target application. As a result, the PPG EduKit can be used in educational activities related to wireless sensor networks.

### 4.3. On-Board Display

This is a general-purpose built-in display Adafruit 128×64 OLED Feather Wing, which has 3 configurable push-buttons and it communicates via I2C. Its goal is to display the raw PPG signal(s) and/or one of the applications of the PPG EduKit such as the computed HR and/or SpO${}_{2}$.

### 4.4. Power

One of the conditions in the selection of compatible embedded platforms to act as digital back-end is the power supply. The analog front-End must be powered with 3V3 and only to be used with MCUs whose logic voltage is 3V3 to avoid system damage. The opamps used are powered with 3V3 in single-supply mode. at the positive supply. This means that the opamps amplification range is from 0 V to 3V3. Signals below or above this range will be seen as a 0V value or 3V3, respectively. The different conditioning stages are not connected to GND but to a virtual ground (VG) = 3V3/2, which equals to 1.65 V. This is done so that the PPG signal will have its reference in the mid-point of the opamp amplification range.

## 5. Platform Evaluation

**Figure 16:**
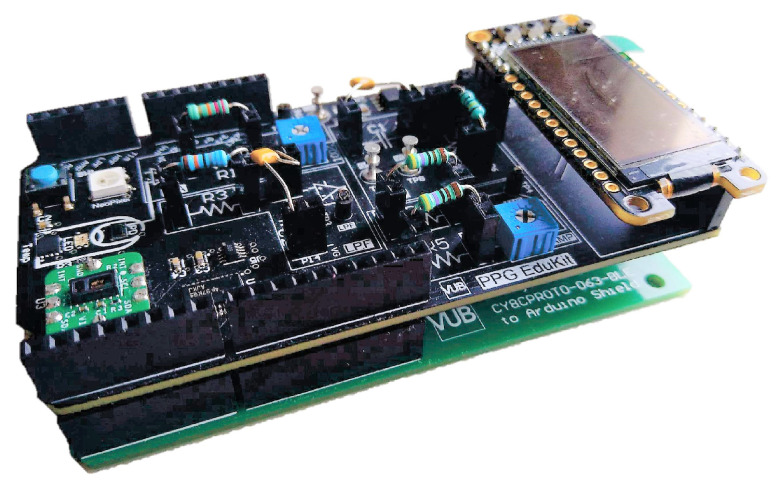
PPG EduKit setup used for the experiments. The digital back-end is a CY8CPROTO-063-BLE Prototyping board using a bridge board to interface the analog front-end.

**Figure 17:**
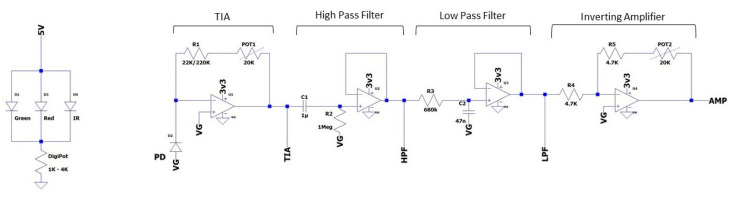
Analog circuit used to test the PPG EduKit performance. A 22 KΩ resistor at the TIA is used for the acquisition with red and IR wavelength, replaced by a 220 KΩ when green wavelength is used. The TIA is followed by a band-pass filter and a inverting amplification of 20 dB (potentiometer set at 10 KΩ).

In this section, the PPG Edukit is evaluated for two common applications of PPG such as HR and SpO${}_{2}$ calculation. Measurements are taken using both the adjustable PPG front-end together with the commercial MAX30101 built-in module, which serves as a comparison. The PPG EduKit used to perform the experiments (Figure 16) includes a CY8CPROTO-063-BLE Prototyping board acting as a digital back-end and using a bridge board to interface the analog front-end. Firstly, the flexibility that this educational kit provides is exemplified through the evaluation of different signal conditioning stages by testing some analog filters, digital filters and their combination. For instance, Figure 17 depicts one possible circuit for the PPG signal conditioning. Secondly, the impact of the selected filters for the calculation of the HR is shown. Finally, SpO${}_{2}$ is calculated through different methods and compared to a reference device. The MAX30101 raw PPG signal, HR and SpO${}_{2}$ are also acquired, serving as a reference.

### 5.1. Filter Analysis

Nowadays, PPG signal processing is an area of research which is driven by the advancements of machine learning in embedded systems, namely the assessment of cardio-vascular diseases such as arterial stiffness and atherosclerosis. PPG signals are often contaminated by high-frequency noise, while the frequency range of interest is typically between 0.5 Hz and 5 Hz [14]. To ensure high-quality PPG signals, the noise impact must be minimized using analog and/or digital filters. Different types of filters and cutoff frequencies can be found in the literature. The authors in [44] describe the impact of different filters in terms of filtering-induced time shifts in PPG signals, while in [45], the authors concluded that the Chebyshev II filter improves the quality of the short-lived PPG signal more effectively than other filters and that the optimal order for the Chebyshev II filter is the fourth order. If the intended purpose of the application is to extract the geometric pulse feature points (first derivative, second derivative, timing characteristics) from the signal, the filter type and the cutoff frequencies should be carefully chosen.

The proposed PPG EduKit includes an adjustable analog module. In Table 1, two examples of possible analog circuit implementations for raw signal conditioning are shown. The difference between both circuits is the bandwidth of the band-pass filter, which is adjusted by the simple change of the resistor R3 that determines the frequency response of the low-pass filter depicted in Figure 17.

Moreover, the adjustable analog filters can be compared to an equivalent digital version implemented in software. For instance, Finite Impulse Response (FIR) filters with the same cutoff frequencies can be used. Most of the frequency content of the PPG signal is below 15 Hz. In order to compare analog and digital solutions, analog band-pass filters with a pass band from 0.5 Hz up to 10 Hz are configured, as well as FIR equiripple filters with an equal pass band. The band-pass filters are good enough to remove unwanted high-frequency noise while keeping the PPG signal components. The main disadvantage of FIR filters is the additional computation power at the digital stage required for the discrete convolution operation.

**Figure 18:**
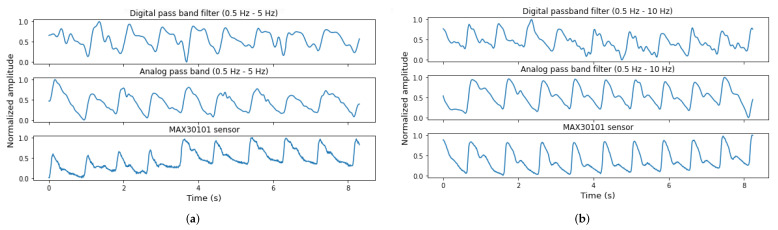
Normalized PPG signals acquired with the PPG EduKit adjustable front-end using both the analog and digital modalities of Filter 1 and Filter 2 configurations. IR light and sampling frequency of 100 Hz are used. All signals in (**a**,**b**) are, respectively, acquired simultaneously. The MAX30101 output serves as a reference. (**a**) Analog and digital Filter 1 with 0.5–5 Hz bandwidth. Outputs of the digital filter (**top**), analog filter (**middle**) and MAX30101 sensor (**bottom**). (**b**) Analog and digital Filter 2 with 0.5–10 Hz bandwidth. Outputs of the digital filter (**top**), analog filter (**middle**) and MAX30101 sensor (**bottom**).

Figure 18 shows the outputs of Filter 1 and Filter 2 in both their analog and digital modalities. Both filters have a number of 396 coefficients, transition bands of 0.5 Hz, an attenuation of 40 dB in the first stop band, respectively, 60 dB for the second stop band. The first filter has a cutoff frequency of 0.5 Hz and 5 Hz, while the second has a cutoff frequency of 0.5 Hz and 10 Hz. The sampling frequency used is 100 Hz.

Both digital filters have a major impact on the PPG wave morphology and cannot be used for geometric feature extraction. However, the output signal would still be valid to extract HR or respiration rate.

### 5.2. Heart Rate

The analog and digital filters can be evaluated in terms of signal quality, either for obtaining a reliable pulse rate (identifiable pulse peaks) or by the reliability of extracted features (identifiable systolic and diastolic waves). Open source tools such as HeartPy [46] can be used to analyze the signal quality by measuring the HR or PhysioNet Toolbox [47] for PPG feature extraction.

Band-pass filters are compared in terms of reliable pulse rate, having as reference the MAX30101 sensor. The IR PPG channel is used for the signals acquisition, while the analog Filter 2 output is obtained by reading the output of the inverting amplifier stage, the digital Filter 2 uses as input the output of the TIA. For the hybrid Filter 2, the signal is read after the analog high-pass filter to then apply a digital FIR low-pass filter. The PPG signal from the MAX30101 sensor module is read over I2C.

**Figure 19:**
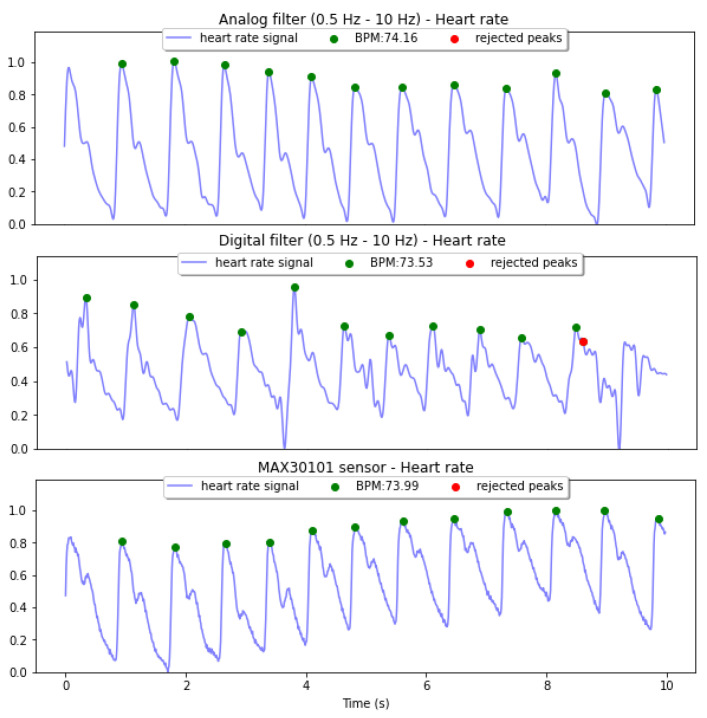
Heart Rate calculation using HeartPy of normalized and filtered signals after the analog Filter 2 (**top**) and the digital Filter 2 (**middle**), using the output of the MAX30101 sensor (**bottom**) as a reference. All the signals are measured simultaneously.

**Figure 20:**
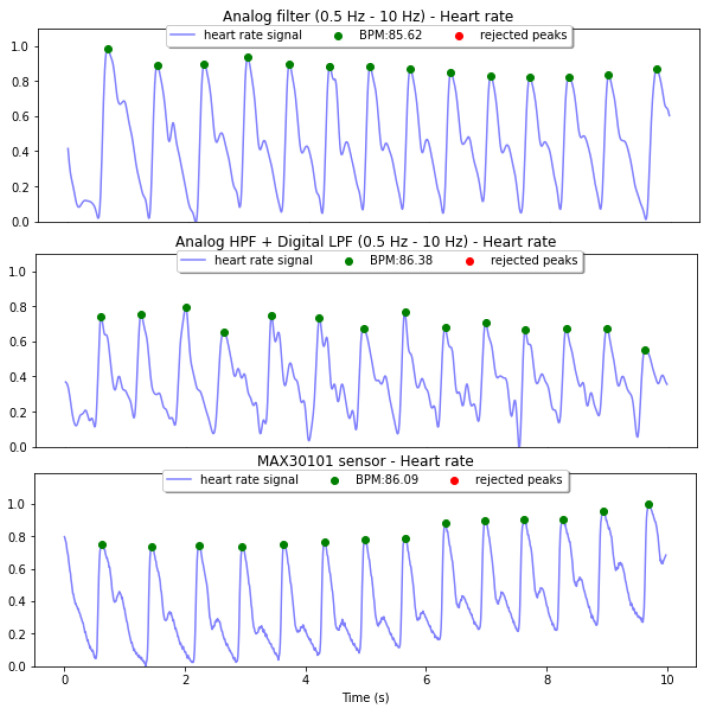
Heart Rate calculation using HeartPy of normalized and filtered signals after the analog Filter 2 (**top**) and the hybrid Filter 2 (**middle**), using the output of the MAX30101 sensor (**bottom**) as a reference. All the signals are measured simultaneously.

Figure 19 and Figure 20 show the acquisition results. Green dots indicate the peaks selected by the HeartPy algorithm to compute HR. Red dots correspond to detected peaks that are rejected for the calculation. The averaged HR, beats per minute (BPM) are also shown for each signal. Comparing the quality of both filters in terms of reliable HR, both methods have as output a PPG signal with identifiable pulse peaks and the HR value is close to the real value obtained from the sensor module. Nevertheless, the signal quality in terms of extracted features reliability of the digital filter is low, since the systolic and diastolic waves are distorted.

Depending on the hardware or software constraints and the outcome of the PPG acquisition, a hybrid approach can be an alternative application.The signal can be filtered using an analog high-pass filter and a digital low-pass filter. As observed in Figure 20, in terms of pulse shape, the signal quality using a hybrid approach is higher than when only the digital filter is used.

### 5.3. Blood Oxygen Level

In this section, the PPG EduKit is used to estimate SpO${}_{2}$ values. Three different methods are used, two in the time domain (a curvilinear approximation and a calculation based on a look-up table) and one in the frequency domain (Fast Fourier Transform (FFT) analysis using a curvilinear approximation). For benchmarking purposes, the built-in MAX30101 sensor is also used in addition to CONTEC CMS50D pulseoximeter, which serves here as a SpO${}_{2}$ gold standard.

The SpO${}_{2}$ measurement is based on time multiplexing the red and IR wavelengths. If both wavelengths are active simultaneously and illuminating the same tissue surface, the measurement can not be done since both will interfere with each other. The LEDs must be switched on and off at a frequency ranging from few hundreds to few thousands of Hz. The ADC sample is taken alternately during the middle of the pulse period of each LED, thus avoiding adding a dead band period between the switching pulses of the LEDs. As previously described in Section 2, the SpO${}_{2}$ can be computed by getting the ratio R at two different wavelengths (red and infrared). This can be achieved in two ways: by measuring the signal AC and DC components in time domain or in frequency domain.

Frequency domain analysis in less prone to artifact interference, leading to a more accurate SpO${}_{2}$ measurement [48]. The only drawback is that the method requires longer signal windows to accurately detect the spectral information, since the FFT is dependent on window size and bin width.

The frequency analysis of the PPG waveform is analyzed in [49] using a FFT hamming window and a 93.75% window overlap. The frequency spectrum exhibits a peak at 0.195 Hz and represents the respiratory frequency, while around 1.44 Hz there is another peak that represents the cardiac pulse. The amplitude density of both components is used to compute R for IR and red wavelengths. In [50], the authors use a 5 s window size to analyze the spectral components. In the frequency domain, these peaks can be respectively considered the DC and AC components of the PPG signal.

**Figure 21:**
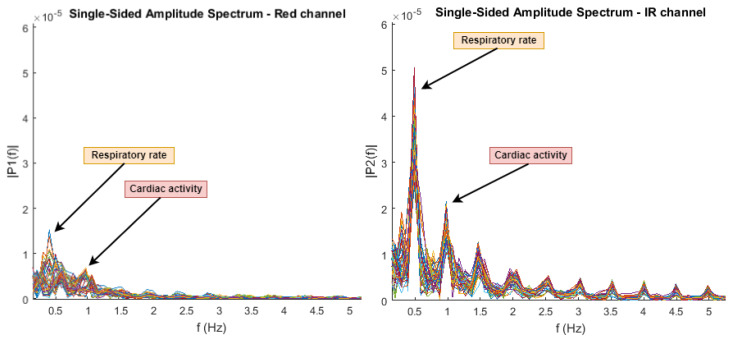
The spectrum amplitude of red (**left**) and IR (**right**) channels using a FFT hamming window. The spectrum amplitude of red (**left**) and IR (**right**) channels is used for the SpO${}_{2}$ calculation. The peaks corresponding to the respiratory rate and cardiac activity are labelled.

The amplitude spectrum of red and IR channels using the PPG EduKit is shown in Figure 21. The sampling rate is 125 Hz, the window size is 310 samples with a 93.54% window overlap. Notice that the IR wavelength has a higher amplitude density and has a higher perfusion index, which is the ratio of AC and DC components expressed as percentage. By finding the peaks of the respiratory rate and cardiac activity, the perfusion index can be calculated for both wavelengths and the SpO${}_{2}$ can be approximated.

To calculate R in the time domain (Equation (1)), the DC component is computed by using a straight-line approximation between two valleys points. Finding the peak value between two valleys, allow us to find a line perpendicular to the line between the two valleys points. The intersection point is the DC value of the signal. The AC component is the distance between the peak value and the DC value.

Once the ratio of both perfusion indexes are computed, a curvilinear approximation or a lookup table can be used to determine the SpO${}_{2}$. For a curvilinear approximation, the “R-curve” is unique for each manufacturer and is based on co-oximeter measurements in a clinical laboratory. To compute the SpO${}_{2}$ Maxim Integrated offers a lookup table and a best-fit line approximation [51].

(3) $${\mathrm{SpO}}_{2}=104-17R$$

**Figure 22:**
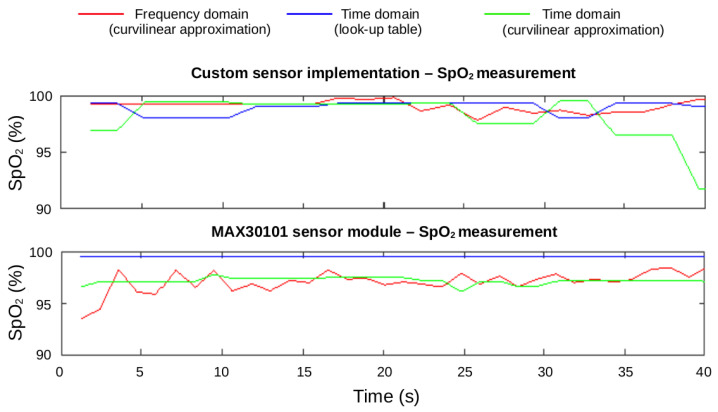
Oxygen saturation comparison between the MAX30101 sensor output and the adjustable analog front-end output of the PPG EduKit using different methods: Frequency domain using the curvilinear approximation (red), time domain using a look-up table (blue) and time domain using the curvilinear approximation (green). The CONTEC CMS50D pulse-oximeter has been also used during the measurements, serving as a gold standard. The reference value measured is 97%.

The SpO${}_{2}$ values are computed from the R (Equation (2)), which is calculated in both the frequency and time domains. For our analysis, the curvilinear approximation is used for both domains, while the lookup table provided by Maxim Integrated is exclusively used in the time domain. These SpO${}_{2}$ values are shown for a window size of 40 s are shown in Figure 22. The PPG signal is filtered using a digital low-pass filter.

In Figure 22, a CONTEC CMS50D pulse oximeter [52] is used as a reference to compare the SpO${}_{2}$ values obtained for the MAX30101 and the PPG signal acquired throught the adjustable PPG analog front-end using both the time-domain method based on a lookup table approach.

## 6. Discussion

The different uses of the PPG EduKit have demonstrated its flexibility to extract the PPG signal. Different analog and digital filters can be applied based on the desired characteristics of the raw PPG signal to be preserved. Two physiological parameters have been extracted and compared against the results reported by the built-in MAX30101, a commercial PPG module, which can also be used as a standalone sensor. Moreover, different methods have been used, demonstrating how the PPG EduKit can be used to explore alternative algorithms for PPG signal analysis or physiological parameter extraction.

In Section 3, an overview of some commercially available devices used to extract and study the photoplethysmographic signal is provided. The PPG EduKit has shown big advantages over these devices if an educational approach is sought. Not only it allows the user to fully customize the different fundamental elements needed for PPG extraction but it also provides a practical tool for the user to fully understand the impact each element has on the final PPG signal. We have provided some application examples, but given its open-hardware and open-software nature, we hope to see additional implementations in the future.

### 6.1. Educational Applications

This section describes the educational potential the PPG EduKit has. The kit has already been used in several courses at the Vrije Universiteit Brussel, with a big acceptance rate by the students. Labs related to applications of PPG, development for embedded systems and wireless communications have incorporated this educational device. Different disciplines can benefit from the PPG EduKit.

- **Electrical Engineering**: The PPG EduKit can be used as an introduction to embedded systems, where all the different stages of the adjustable analog front-end together with the digital back-end can be implemented.
- **Computer Science**: A wide variety of signal processing algorithms can be applied to the raw PPG signal extracted from the EduKit.
- **Telecommunication Engineering**: The PPG EduKit used with a digital back-end that allows wireless communication can be used to introduce networks and connectivity in health care. For instance, they can be integrated as nodes in wireless sensor networks.
- **Data Science and Machine Learning**: The PPG EduKit can be used as an acquisition system where signal quality assessment or HR/SpO${}_{2}$ calculation can be implemented by using machine learning, among other techniques.
- **Biomedical Engineering**: Biomedical Engineering students can benefit from the PPG EduKit accessing raw PPG signals or by adjusting the system for the extraction of physiological parameters, among other applications.
- **Health Care Sector**: Medicine or nursing students, among other medical disciplines, can use the PPG EduKit as a practical introduction to PPG, where the technology can be understood in detail.

### 6.2. Future Work

The PPG EduKit is an open hardware solution, which can be replicated by other institutions, teachers, students or hobbyist. One of its limitations are the reduced options for analog filtering. The filtering modules only allow the implementation of first order RC filters. Nevertheless, this limitation can be overcome by using the through-hole female headers of the different stages as input/output of external circuits (in a breadboard), such as more complex filters, DC tracking or ambient light cancellation, among others. This can lead to a maximization of PPG EduKit capabilities in the form of alternative workshops/labs that reach a broader spectrum of engineering/medical students.

The PPG EduKit could be combined with an ECG acquisition module, which would extend the scope of this solution in the biosignals domain. In addition, the flexibility provided by the PPG EduKit would facilitate the integration of other modules such as temperature, force and inertial sensors. This would allow students to observe and analyze the influence of the main phenomena that influence the quality of the PPG signal.

The PPG Edukit PPG could be adapted to be compatible with 5 V logical-level devices to extend the range of compatibles microcontrollers. In addition, a better integration with Mixed-Signal ICs, like PSoCs, would allow the creation of an adjustable wearable. Such a device could be used for the implementation and characterization of additional analog filters, embedded signal processing techniques, ambient light compensation circuits or DC tracking, among others. Overall, these additional features would enlarge the PPG EduKit scope.

## 7. Conclusions

The grown interest in PPG technology demands novel educational tools to facilitate the learning, understanding and exploration of this technology. The commercial solutions, while covering important applications of PPG technology, are not sufficient as educational tools. The here presented PPG EduKit is an educational platform intended to address the needs of engineering and medical students, with a special focus on biomedical engineering programs. The PPG EduKit is the first PPG technology reported in the literature with an exclusive educational approach. It is designed to give the user full contrsol and understanding over the different steps needed to acquire, condition, visualize and process the raw PPG signals used to extract meaningful physiological information. The modular approach of the platform facilitates the support of different embedded devices to perform digital signal processing operations needed for all type of PPG-based applications. Our evaluation has shown how the PPG EduKit supports multiple configurations to extract relevant physiological parameters such as heart rate and SpO${}_{2}$ extraction.

## Figures and Tables

**Table 1:** Values of the passive components needed to generate two different analog band-pass filters (Analog Filter 1 and Analog Filter 2) in the adjustable analog front-end.

Filter	R1	C1	R2	R3	C2	R4	R5
Analog Filter 1 (0.5–5 Hz)	22 kΩ	1 μF	1 MΩ	680 kΩ	47 nF	4.7 kΩ	4.7 kΩ
Analog Filter 2 (0.5–10 Hz)	22 kΩ	1 μF	1 MΩ	330 kΩ	47 nF	4.7 kΩ	4.7 kΩ

## Data Availability

The PPG EduKit is designed to be an open source platform. Schematics of the PCBs and the source code used to perform the experiments are available online: https://gitlab.com/etrovub/wearables/publications/ppg-edukit.
